# Microdroplet Sandwich Real-Time RT-PCR for Detection of Pandemic and Seasonal Influenza Subtypes

**DOI:** 10.1371/journal.pone.0073497

**Published:** 2013-09-16

**Authors:** Stephanie L. Angione, Zintis Inde, Christina M. Beck, Andrew W. Artenstein, Steven M. Opal, Anubhav Tripathi

**Affiliations:** 1 School of Engineering, Center for Biomedical Engineering, Brown University, Providence, Rhode Island, United States of America; 2 Division of Biology and Medicine, Brown University, Providence, Rhode Island, United States of America; 3 Department of Medicine and the Center for Biodefense and Emerging Pathogens, Baystate Health, Springfield, Massachusetts, United States of America; 4 Warren Alpert Medical School, Brown University, Providence, Rhode Island, United States of America; 5 Memorial Hospital of Rhode Island, Pawtucket, Rhode Island, United States of America; Northeastern University, United States of America

## Abstract

As demonstrated by the recent 2012/2013 flu epidemic, the continual emergence of new viral strains highlights the need for accurate medical diagnostics in multiple community settings. If rapid, robust, and sensitive diagnostics for influenza subtyping were available, it would help identify epidemics, facilitate appropriate antiviral usage, decrease inappropriate antibiotic usage, and eliminate the extra cost of unnecessary laboratory testing and treatment. Here, we describe a droplet sandwich platform that can detect influenza subtypes using real-time reverse-transcription polymerase chain reaction (rtRT-PCR). Using clinical samples collected during the 2010/11 season, we effectively differentiate between H1N1p (swine pandemic), H1N1s (seasonal), and H3N2 with an overall assay sensitivity was 96%, with 100% specificity for each subtype. Additionally, we demonstrate the ability to detect viral loads as low as 10^4^ copies/mL, which is two orders of magnitude lower than viral loads in typical infected patients. This platform performs diagnostics in a miniaturized format without sacrificing any sensitivity, and can thus be easily developed into devices which are ideal for small clinics and pharmacies.

## Introduction

Influenza is a major pathogen of humans and causes significant respiratory infections, resulting in 250,000 to 500,000 deaths worldwide annually [1], [2], [3]. The influenza virus is a member of the *Orthomyxoviridae* family which includes influenza A, B, and C viruses [4]. Influenza A viruses are of the most concern to public health and are responsible for the yearly seasonal flu epidemics as well as global pandemic events characterized by high morbidity and mortality [5]. The World Health Organization assigns names to each virus strain with an antigenic description of the hemagglutinin (HA) and neuraminidase (NA) surface proteins such that there are 13 different HA types and 9 NA types among influenza A viruses. Antigenic variation (i.e. “antigenic drift”) in the HA and NA proteins is responsible for most of the seasonal variation in influenza A viruses.

The infrequent occurrence of profound alterations in these surface antigens (i.e. “antigenic shift”) gives rise to influenza virus strains that are essentially unrecognizable to the human immune system, leading to the possibility of global pandemic events [6], [7], [8], [9].

Currently, hospitals, small clinics, and other community health care assets often rely on rapid immunoassay diagnostics to identify influenza. Such assays are typically associated with lower sensitivity (39–62%) than gold standard methods such as RT-PCR [10], [11]. During the 2009 pandemic, the shortcomings of the rapid antigen tests were particularly apparent, with reported sensitivities for detecting the pandemic strain as low as 25% [12]. At such low sensitivities, these tests lose their diagnostic relevance. Other tests, like viral culture and direct fluorescent antigen (DFA) testing are more robust and reliable in terms of sensitivity, but are time consuming and often difficult to perform [10]. Thus, RT-PCR has emerged as a potentially better option for adoption and integration as a rapid test for influenza detection. It possesses distinct advantages over other modalities in that it provides clinicians with subtyping information and boasts sensitivities in excess of 90% [10], [11], [13]. RT-PCR typically identifies influenza strains based on differences in genetic composition through the discriminate use of specifically designed primers. This diagnostic approach has proven effective in clinically identifying influenza strains, including those responsible for the 2009 outbreak of swine-origin influenza [14], [15]. Further advances in this approach have included the use of real-time RT-PCR (rtRT-PCR) to enhance the ability to detect both the target sequence and quantify the number of viral particles present.

The specific characteristics of influenza, particularly its ability to mutate and spread rapidly, necessitate improved diagnostic technologies which can quickly pinpoint an epidemic at its source since antiviral treatments, isolation, and other containment strategies must be implemented promptly in order to prevent broader infection [16], [17], [18]. The platform presented here produces results in real time, allowing for quantification of viral load and subtyping to improve treatment and enhance epidemic surveillance. Microfluidic approaches are useful in addressing these issues, specifically by incorporating the elements of traditional RT-PCR into a platform in which the reactions take place in low volume quantities [18], [19], [20]. While there are several microfluidic methods that provide a positive/negative identification of influenza, there are only a few examples in the literature that utilize a microfluidic platform for nucleic acid subtyping and one which utilizes human patient derived influenza samples of circulating strains using a different amplification method known as loop-mediated isothermal amplification (LAMP) [21], [22], [23], [24]. Thus, to our knowledge, our assay and device is the first to subtype clinically derived influenza strains of epidemiological relevance on a microfluidic apparatus using a gold standard method like rtRT-PCR. We believe that this marks a significant step towards providing a platform for subtyping circulating influenza viruses and this approach promises to help facilitate more sensitive and rapid testing for influenza and other diseases [25].

Here we present a droplet sandwich platform for the differentiation of influenza strains using rtRT-PCR. We demonstrate that the platform can effectively differentiate between H1 and H3 strains of influenza, as well as between swine-origin and seasonal strains of H1. Detection using this platform is highly sensitive, amplifying clinical samples as dilute as 10^4^ copies/mL. The platform is robust, using a simple apparatus, and producing results which are reproducible and generalizable to other diseases, including HIV and bacterial strains. Thus, the development of this platform represents an important step towards improved diagnostic technologies for influenza, other infectious diseases, as well as broader applications requiring PCR.

## Methods

### Primer Design and RT-PCR

Primers were designed using consensus sequences from the online NCBI Influenza Virus Sequence Database (http://www.ncbi.nlm.nih.gov/ genomes/FLU/Database) and obtained from Integrated DNA Technologies (IDT, Coralville IA). The sequences were sorted using a house-written MATLAB program to identify short sequences of the highest conservation for each subtype from the coding region of the hemagglutinin segment. The subtypes utilized were H3, H1p, and H1s. Additionally, due to the high similarity of consensus sequences between H1p and H1s lineages, primers were specifically chosen to have only high conservation within one of the H1 subsets. Lastly, the primer choices for H1p or H1 s were compared to the corresponding sequence in the opposing subset to ensure that minimal sequence similarity was present. For each primer pair, primers were optimized to anneal between 50–55°C. The self- and hetero-dimerization energies of each of the primer pairs were evaluated in silico with DINAMelt (DINAMelt Server, RNA Institute, SUNY Albany, 75 Albany, NY; available at http://mfold.rna.albany.edu) and Primer3 Plus (http://www.bioinformatics.nl/cgi-bin/primer3plus/primer3plus.cgi/) to ensure limited primer-dimer formation. Several candidate primer pairs for each gene were ordered from IDT. Multiple iterations of primer design and validation of the primers with the PCR platform were run to identify the optimum grouping of three primer pairs to utilize on the platform. Table 1 shows the sequences of the primers developed. The sensitivity and specificity of each primer set are displayed in Table 1 which displays the proportion of true positives correctly identified and the number of true negatives correctly identified. Non-platform controls were performed for all samples tested in order to verify the sensitivity and specificity of the primers. These consisted of a 10 µl reaction mix from the Superscript III reverse transcriptase and Platinum Taq DNA polymerase kit (Life Technologies, Carlsbad, CA). Both reverse transcription and PCR were carried out in the same tube or on the droplet sandwich platform. This included 1× Taq buffer, 0.2 mM dNTPs, 1.5 mM MgCl_2_ and 0.2 µM of both the forward and reverse primers. SYBR Green I (Invitrogen, Carlsbad, CA) was utilized for fluorescence detection on the platform at a concentration of .1×. Cycling consisted of a 30 minute RT step at 50°C, followed by 40 cycles of 94°C for 15 seconds, the primer-specific annealing temperature (see Table 1) for 15 seconds, and 68°C for 30 seconds. An initial denature was done following the RT step for 15 minutes for off-chip controls using a thermal cycler (Biorad Technologies, Hercules, CA) and 2 minutes for platform droplet amplification. The size of products were determined using DNA 1000 chips on an Agilent 2100 Bioanalyzer (Agilent Technologies, Santa Clara, CA).

**Table 1:** Primer sets and sensitivities for each subtype.

Oligonucleotide	Sequence information	Additional information
**Influenza H3 RT-PCR primer set**	235 bp, Annealing temperature: 52°	Sensitivity: 95%, Specificity: 100%
Forward primer	5′-AATTTTGATGCCTGAAACCGTACCA-3′	25 nt
Reverse primer	5′-ACTCCAAATGGAAGCATTCCCAATG-3′	25 nt
**Influenza H1s RT-PCR primer set**	179 bp, Annealing temperature: 51°C	Sensitivity: 89% Specificity 100%
Forward primer	5′-TCTGTAGTGTCTTCACAT-3′	18 nt
Reverse primer	5′-CTCTACTCAGTGCGAAAG-3′	18 nt
**Influenza H1p RT-PCR primer set**	167 bp, Annealing temperature: 49°C	Sensitivity: 97% Specificity 100%
Forward primer	5′-ATGCTGGATCTGGTATTAT-3′	19 nt
Reverse primer	5′-CAATTTTGTGCTTTTTACATATT-3′	23 nt

### Spiked Sample Preparation

Spiked viral isolates were initially obtained from Charles River Laboratories (Wilmington, MA). The viruses were maintained in Madin Darby Canine Kidney (MDCK) cells, and stock suspensions were quantified by serial ten-fold dilutions in tissue culture media and then determination by TCID50 assays in MDCK cells. The virus concentrations were prepared by heat-inactivation. The specific strains were Influenza A (H1N1)/PR 8/34 and Influenza A (H3N2)/Aichi/68 for the H3 and H1s subtypes.

### Viral RNA Production

For the rtRT-PCR standard curve, synthetic H3 viral RNA was produced. A wild-type Influenza A H3 DNA sequence, NCBI AF348176 (A/HongKong/1/68(H3N2)), coding for the hemagglutinin segment was synthesized by DNA 2.0 (Menlo Park, CA), inserted into a pJ10 *E. Coli* propagation plasmid, and sequenced. A truncated SP6 RNA polymerase promoter, which lacks a G at position 1, was followed by the reverse compliment of this H3 cDNA sequence. The 3′ end of the sequence included a restriction site for Kpn2I, which cleaves the sequence ‘TCCGGA’ one base in from the 5′ ends on both strands. Plasmid cleavage with this enzyme produced a 3′ overhang that was removed using the Klenow fragment of DNA polymerase I. The SP6 transcription produced H3 viral RNA with native 5′ and 3′ ends. The restriction digest was separated on an Agilent 7500 DNA chip. Transcription products were visualized on an Agilent 6000 Nano RNA microchip (Agilent Technologies, Santa Clara, CA). The in-vitro transcribed RNA was reverse transcribed to cDNA, inserted into a plasmid, and sequenced to confirm the product was correct. These plasmid-derived DNA sequences were used for generating non-pathogenic viral RNA and referred to in this paper as vRNA.

### Collection of clinical samples

Nasopharyngeal swab samples from 40 distinct patients were randomly selected and clinically de-identified for use in this study. These clinical samples were drawn from existing laboratory specimens that had been collected at Memorial Hospital of Rhode Island for clinical indications during the 2010 influenza season. The study was given exemption from the Memorial Hospital Institutional Review Board (IRB) and informed consent was not required as the samples utilized were randomized and de-identified following routine clinical evaluation.

### Isolation of viral RNA from clinical samples

Due to the complexity of nasopharyngeal swab samples, isolation of nucleic acids was necessary to remove proteins and various other particles. This was done using the MagMax Viral Isolation kit (Life Technologies, Carlsbad, CA). Following the isolation procedure the extracted RNA concentration was determined using a Nanodrop Spectrophotometer (Thermo Scientific, Wilmington, DE). The concentrations of the extracted samples are provided in Table S1.

### Validation of RT-PCR results with antigen detection and viral culture

The results of both the original screening step for sample collection, the influenza rapid antigen test as well as the results of our unique RT-PCR assay were compared and validated utilizing clinical assays. Rapid influenza nucleoprotein antigen detection was performed using an immunochromatographic assay according to the manufacturer's instructions (BinaxNow/influenza A and B). Primary viral isolation and propagation was done under biosafety level 2 conditions using MDCK cell culture methods as previously described [28]. Sensitivity, specificity, PPV, NPV and accuracy calculations were done using standard methods.

## Results

### Droplet sandwich platform

Droplet PCR is an advantageous alternative to traditional benchtop PCR since the use of small sample volumes allow for faster heat transfer and thermal equilibrium due to decreased diffusion distances. In traditional PCR, thermal diffusion distances are on the length scale of 0.2 mL tubes, which are∼10.0 mm for the longest length versus 2.8 mm for the droplet format. The thermal diffusion time scale in water, 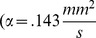, using 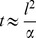, is more than 10 times greater for conventional PCR than the droplet method. Additionally, droplet PCR formats are particularly useful for amplifying DNA in small volumes in that droplets act as discrete reaction vessels, facilitating faster temperature cycling and reaction times as well as limiting sample carryover and contamination.

**Figure 1:**
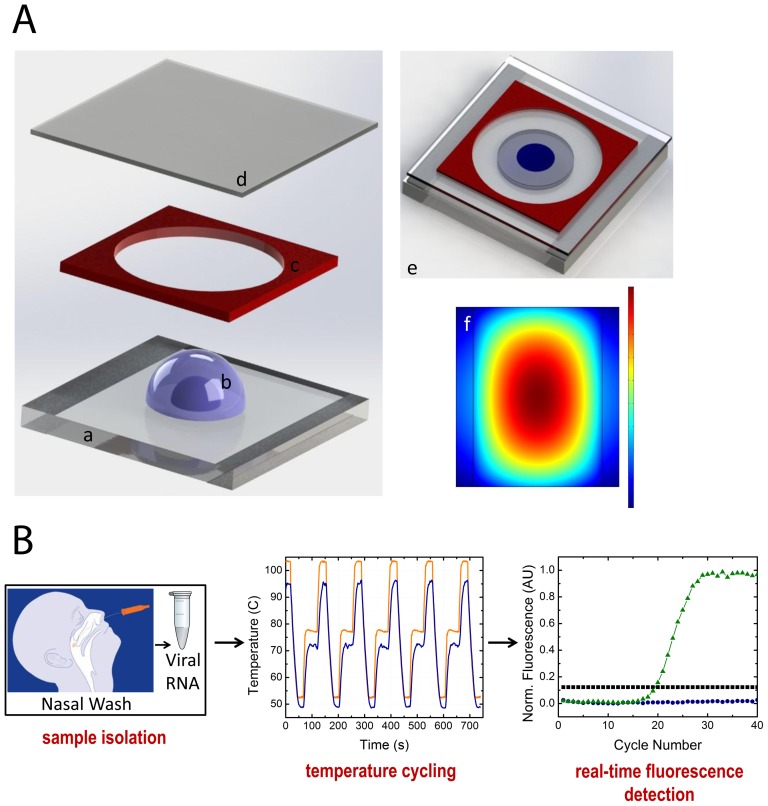
Droplet sandwich platform. a: Drawings of platform: 3D drawing of the droplet sandwich platform displaying the ITO coated glass (a) with a compound droplet (b) surrounded by a spacer (c) and covered with a coverslip (d), which is fully assembled to sandwich the compound droplet in a reaction chamber (e). The ITO surface heats radially, as displayed the modeled heating profile for the ITO glass when 15 V is applied to the resistive surface as generated by COMSOL Multiphysics (f). The dimension of the slide is 40 mm×40 mm and the compound droplet is approximately 2.8 mm in diameter. b: Workflow and representative data: Sample isolation is done from nasopharygeal swabs and the rtRT-PCR mix is transferred to the droplet sandwich platform for thermal cycling. Temperature cycling occurs at the center of the radial profile as displayed by the plot where the black line represents the controlled surface temperature and the red line is the calibrated droplet temperature. Fluorescence is collected in real-time during the extension phase of PCR, with DNA amplification of positive samples displayed in green, negative samples with no change over time in blue and calculated threshold in black.

The sandwich platform consists of a 2 µl droplet of RT-PCR mix surrounded by mineral oil, producing a disc shaped compound droplet. Indium tin oxide (ITO) coated glass provides heating for RT-PCR, and an imaging spacer and a cover slip prevent evaporation during cycling (Fig. 1a). Optically clear Fluorinated Ethylene Propylene (FEP) tape is used as the reaction surface for each experiment to prevent adsorption of reagents, as well as, provide a disposable surface between experiments. In this way the ITO glass is used as a platform for many PCR reactions and doesn't require replacement as the tape is the disposable element of the design. A proportional-integral-derivative (PID) feedback element controls the droplet temperature and can be programmed to match the cycling conditions for a chosen RT-PCR protocol. The resistive thin film heats rapidly and radially, with the temperature feedback system focused at the center with the droplet (Fig. 1a). Both temperature and fluorescence signals are collected simultaneously. The temperature profile (Fig. 1b) displays the temperature cycling achieved using the droplet platform at the center of the radial heating profile. A specific advancement of our system is that it only requires a very low input voltage for operation (∼15 V) and also utilizes a micro-fan for cooling. The temperature cycling program displayed is 95°C for 15 s, 49°C for 15 s and 72°C for 30 s. The average cycle time is approximately 100 s, and the ramp rate for heating is ∼3.0°C/s and cooling is ∼2.0°C/s. The ramping rates displayed can be further optimized to decrease the running time for the PCR reactions as the time required to reach thermal equilibrium is reduced in the droplet format. A similar platform was employed for amplification of model DNA and analysis of the system [19]. This platform can be integrated with battery powered detection and heating and USB linked software for control and data acquisition.

This platform provides a reliable, real-time diagnostic method targeted for detecting infectious diseases down to specific subtypes/strains. Our method couples the unparalleled sensitivity of polymerase chain reaction with a compact, simple, and fast real-time platform that can uniquely identify the specific influenza subtype.

### H3 vRNA serial dilution

**Figure 2:**
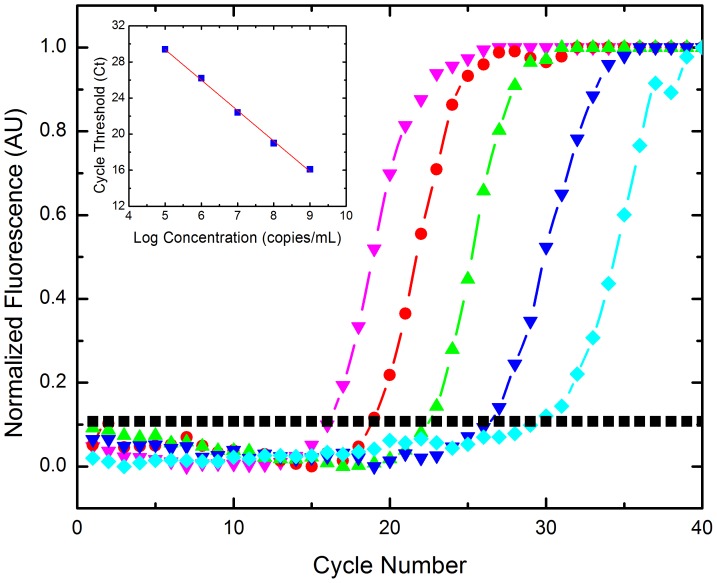
Serial dilution of H3 vRNA of 10^9^−10^5^ copies/mL. Threshold was calculated as 10× standard deviation of the background signal. 10^5^ copies/mL was the lowest concentration amplified using the one-step RT-PCR reaction on the tablet platform. Legend: 10^9^ copies/mL (pink▾), 10^8^ copies/mL (red •), 10^7^ copies/mL (green▴), 10^6^ copies/mL (blue▾), 10^5^ copies/mL (turquoise ♦), threshold (black ▪). Inset: Efficiency plot of H3 vRNA serial dilution series. Displays the Ct values vs. log concentration of vRNA, with a linear regression fit of R^2^ = 0.998. The slope of the line is −3.38, providing an efficiency of 97.6% using E = 10^(−1/slope)^−1 with an average standard deviation of ±1.3 cycles.

To determine sensitivity and evaluate the applicability of the tablet platform as a clinical diagnostic tool, we performed a standard curve dilution of viral H3 RNA (vRNA) to calculate the efficiency and accuracy of the tablet droplet platform for performing rtRT-PCR. A serial dilution of vRNA was evaluated as a template on the real-time platform with starting concentrations of 10^9^, 10^8^, 10^7^, 10^6^, and 10^5^ copies/mL of vRNA (Fig. 2). We determined the sensitivity of the system for this assay to be 10^5^copies/mL of RNA as this was the lowest concentration detected. No template controls (NTC) were also performed and showed no fluorescence change over time.

The corresponding cycle threshold (Ct) values for each of the dilutions was determined by plotting the log linear phase of amplification with the log of the threshold and the Ct values were then plotted against the log of the concentration (Fig. 2 inset). The slope of the linear regression fit was determined to be −3.38, with a correlation of >99%, and an average standard deviation for the calibration curve was determined to be ±1.3 cycles. The efficiency of the system can be calculated using E = 10^(−1/slope)^−1, which was found to be 97.6%. Typical and acceptable efficiencies are generally 90–110% for real-time PCR. The system can perform the one-step RT-PCR reaction as well as conventional real-time PCR systems but more rapidly and with a fraction of the reaction volume. The sensitivity corresponds to a low viral load of 10^5^ copies/mL, as viral loads from the nasopharynx are generally greater than 10^6^ virions/mL. This makes the sensitivity of our platform and assay ideal for detecting RNA from patient samples [26], [27]. The efficacy of the assay is remarkably high as demonstrated by the greater than 97% efficiency.

### RT-PCR typing of clinical samples

Prior to typing clinical samples, each primer set was tested individually against a set of 10 spiked influenza virion samples of unknown subtype in respiratory sample collection media, provided by Memorial Hospital of Rhode Island (File S1). The gel plot (Fig. S1) displays the positive results for the primer pairs, and no cross-reactivity was found between primer sets.

Nasopharyngeal swab samples from 40 distinct patients were randomly selected and clinically de-identified for use in this study. These clinical samples were drawn from existing laboratory specimens that had been collected at Memorial Hospital of Rhode Island for clinical indications during the 2010 influenza season. The samples were evaluated using a rapid antigen test (see Methods) and concurrent viral culture was performed at the Memorial Hospital Infectious Disease Research Laboratory according to a previously published procedure [28]. Workers at both laboratories remained blinded to the results from the other lab. Discrepancies were observed between antigen and culture testing, which will be examined in the following Discussion section.

The clinical isolates comprised nasopharyngeal swab specimens in transport media. Following RNA isolation, each sample was tested for subtype with the primer sets (see Methods). Table S1 displays the subtyping results from 40 samples, including the extracted RNA concentrations for each sample. The table also displays an indication of the agreement with the other diagnostic techniques used to validate influenza infection, including antigen testing and viral culture. Of the 40 samples, 22 were found to be positive by RT-PCR and 18 were negative. The positive samples were either the H3 or H1 swine subtype. None of the positive samples were found to be of the H1N1s seasonal subtype. RT-PCR and viral culture were able to detect two and three positive samples respectively, which were negative for antigen testing. RT-PCR and the antigen assays detected six influenza positive samples that viral culture did not. These results were validated in triplicate by RT-PCR, and de-contamination methods were employed for both no-template controls (NTC) and antigen negative samples (see Methods).

### Viral load determination of influenza A clinical samples

**Figure 3:**
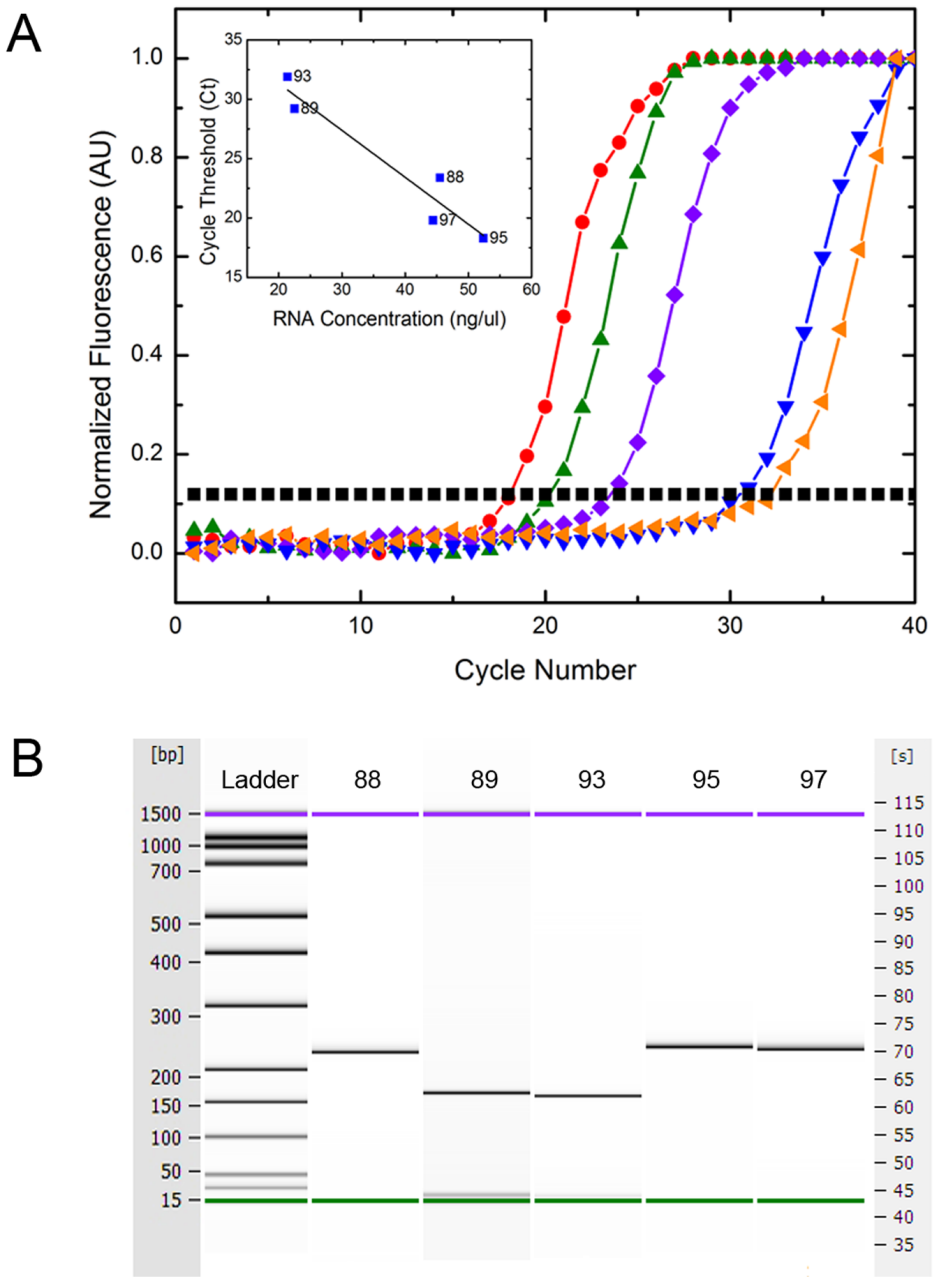
rtRT-PCR results for clinical samples. a: Samples 88, 89, 93, 95 and 97. Displays normalized fluorescence vs. cycle number of 5 clinical positive samples. Of the samples, 89 and 93 were H1p positive and samples 95, 97 and 88 were H3 positive when amplified with those respective primer sets. There was no cross-reactivity with the other primer sets and a NTC displayed no change in fluorescence over time. Legend: 95 (red •), 97 (green ▴), 88 (purple ♦), 89 (blue▾), 93 (orange ◂), threshold (black ▪). inset: Plot of the calculated cycle threshold vs. total RNA concentration following extraction from clinical samples. The linear regression fit of the Ct values is shown by the solid black line with an R^2^ = .956. b: Generated gel plot for samples 88, 89, 93, 95 and 97. Displays the electrophoresis gel image from the Agilent Bioanalyzer 2100 and displays the 235 base pair (bp) amplicon for samples 88, 95 and 97 indicative of the H3 subtype and displays the 167 bp amplicon for samples 89 and 93 indicative of the H1p subtype.

We performed rtRT-PCR of five positive and five negative clinical samples to evaluate the ability of the platform to amplify viral RNA at clinically relevant concentrations as obtained from patients with influenza (Fig. 3a). Samples 88, 95 and 97 were determined to be the H3 subtype by those primers as demonstrated by the gel plots (Fig. 3b) and were negative for H1p and H1s RNA. Samples 89 and 93 were determined to be H1p by those primers, and negative for H3 and H1s (Fig. 3b). Negative samples showed no change in fluorescence over time, and no bands on the electrophoresis gel plots. Directly following isolation of RNA from the clinical samples, the total RNA concentration for each sample was determined, so as to correlate with viral load. The inset displays the cycle threshold (Ct) for each sample as a function of its RNA concentration.

## Discussion

A platform with the ability to sensitively and rapidly detect both H3 and H1 strains and variants is particularly important as demonstrated by the severity of the most recent 2012/2013 flu season. This season was marked by an early start with a major H3N2 strain that hasn't been seen in circulation for at least five years, making most individuals susceptible to infection. Rapid subtyping is of high clinical value in that during some flu seasons, the viral susceptibility to antiviral medications like oseltamivir differs based upon subtype when multiple subtypes are in circulation. One study found that resistance to oseltamivir was more common among seasonal H1N1 virus infections (27%) compared with H3N2 (3%), and rare cases of H1N1 pandemic resistance [29], [30]. Determining the subtype at the outset of symptoms can guide early appropriate therapy, helping to decrease morbidity in the population. Additionally, epidemiological surveillance of subtypes is important in determining if the vaccine fit is sufficient and to evaluate if any antigenic drift has occurred, which would make the vaccine less effective. Our assay with novel H3 primers detects 95% of H3 strains and 97% of H1p strains with 100% specificity, making the diagnostic potential coupled with the speed of our assay unparalleled for influenza diagnostics (Table 1, Methods). Although the sensitivity for the seasonal primers is less ideal at 88.6% (Table 1, Methods) by adding additional primers with single base pair changes, the collective sensitivity can be increased to 94%, which was determined using database sequence analysis of the H1s strains. With the small sample set utilized here, we did not find any discrepancies with subtyping using our primers, but for a larger study it would be more advantageous to utilize several distinct primers for subtyping of the H1s strains so as to ensure adequate coverage of at least 94% of reported sequences.

Since our technique does not have an equivalent single gold standard to provide the same sensitivity, specificity, accuracy and information content, a combination of the methods was used to determine these metrics (Table S1). Any sample which had at least one positive result, from PCR, viral culture or antigen detection was deemed to be a positive. True negatives were thus assumed to have a negative test by all three methodologies. It should be noted that 35% of positive samples were positive by two tests, and 61% by three tests, with only one sample (4%) being positive by only one test. The RT-PCR demonstrated higher sensitivity in that it not only identified all the positive rapid antigen samples, but also identified two rapid antigen negative samples as positive. These two antigen negative but H3 PCR positive samples are likely true positives as indicated by positive culture, which were thus false negatives for the antigen test. From our results, it is clear that the rapid antigen tests were less reliable with this subset of samples, such that 87% of the samples were accurately identified as either influenza positive or negative. Six PCR/Ag positive samples were found to be culture negative, which is likely due to either the use of antiviral medication in those patients or the presence of non-viable viruses in nasal secretions. RT-PCR has the advantage of detecting these non-viable viruses that do not necessarily represent active infection, but indicate the presence of recent past infection. The results of viral culture confirm the utility of this RT-PCR assay, in that we found 82.5% accordance with culture results. The fact that RT-PCR detected more positives than viral culture is not an uncommon result, as culture may miss up to 46% of influenza positive samples, especially in patients with advanced clinical course of disease [31]. One culture positive sample was found to be both PCR and antigen negative, which is likely a true influenza positive sample, giving our assay 96% overall sensitivity. This may have been related to a viral load in this sample that was below the level of detection of our assay, assay inhibition or sample loss. The assay thus had 100% specificity, 100% positive predictive value (PPV,) and 94% negative predictive value (NPV) with an accuracy of 97.5%. By utilizing RT-PCR in place of culture, the diagnostic yield for positive samples increased 29% and with the time for culture requiring on average 5 days, our assay is approximately 60 times faster than culture and provides accurate subtyping of not only H1/H3 but H1p/H1s subtypes [10].

Although our assay has the ability to subtype the H1s type as well as the H1p, none of the samples tested were positive for H1s. This is not surprising as the CDC reported 99.8% of H1N1 viruses tested during the 2010 flu season were H1p, or A/California/7/2009-like [32]. Additionally, of the samples tested, we determined 23% to be H1p and the other 77% to be H3 of the 22 samples that were PCR positive. The CDC reported 38% H1p and 62% H3 as the subtypes for 28,661 influenza A samples for the 2010 season [32]. With a larger sample set, the percentages would skew more towards the CDC values, but our results are consistent with the overall subtype trends demonstrated during the 2010 season. The current tests available through a combination of antigen testing and viral culture cannot compare in sensitivity, specificity, speed or information content as provided by our RT-PCR assay and droplet sandwich platform.

By testing a subset of H3 and H1p positive samples using our unique tablet platform we were able to provide a viral load approximation for the clinical samples tested. Using the standard curve (Fig. 2), the quantities of the unknown samples were then extrapolated based on their Ct values, which are displayed in Table S2. The viral load of the H1p samples were quite low compared to the H3 samples, which could be a result of anti-viral treatment of those patients but is also indicative of the H1p subtype itself, with reported viral loads in the range of 10^5^–10^7^ virions/mL [26]. These results follow the general trend of the total RNA isolation concentrations reported (Fig. 3a) with viral loads ranging from 8.3 to 4.3 log(copies/mL) for samples 95 and 93 respectively. The lowest viral load of 4.3 log(copies/mL) is far below the typical patient viral loads of 6.0 log(copies/mL). This indicates that this patient had a reduced viral load either from late stage infection or antiviral use, but our assay can detect below the clinically relevant threshold, providing high sensitivity in detecting influenza.

## Conclusion

Thus, we have presented a platform and assay that can accurately and sensitively subtype influenza strains of epidemiological relevance. The emergence of swine pandemic H1N1 and the most recent flu season of 2013 has demonstrated the necessity of subtyping influenza for monitoring broad antigenic changes in circulating viruses for better vaccine development and human immunity. Additionally, coupling an accurate and sensitive method like RT-PCR with a small point-of-care focused platform can make detecting and subtyping influenza easier, cheaper, and faster for smaller clinics which often see many flu patients during the peak season but heavily rely on antigen tests which are less informative and accurate. Influenza is one of the many infectious diseases that pose a significant threat to humans; requiring sensitive, specific and rapid diagnosis in order to serve and maintain public health [33]. With the development of our sandwich platform and assay, detection and monitoring of infectious diseases from both viral and bacterial origin can be improved to better the health of a wide variety of communities both in the US and around the world.

## Supporting Information

Figure S1 **Gel Plot of H3 and H1-seasonal Spiked Samples.** Displays the generated gel plot for the series of spiked samples A-K. Samples A, B, D, E, F, G and H display the 230 bp amplicon for the H3 subtype. Samples J and K display the 179 bp amplicon for the H1 seasonal subtype. Sample C is a no template control, and the 50 bp amplicon is a primer dimer associated with the H3 primers. This primer dimer is also visible in some of the lower concentration H3 samples, but it does not interfere with the sub-typing process.
(TIF)

Table S1 **Summary of clinical sample typing.**
(DOCX)

Table S2 **Copy number of influenza viral RNA in clinical samples.**
(DOCX)

File S1 Spiked sample validation of off-tablet controls.
(DOCX)
