# Supplementary figures and images for: Microdroplet Sandwich Real-Time RT-PCR for Detection of Pandemic and Seasonal Influenza Subtypes
Source: PLoS One. 2013 Sep 16;8(9):e73497. doi: 10.1371/journal.pone.0073497 (PMC3774678; doi:10.1371/journal.pone.0073497)

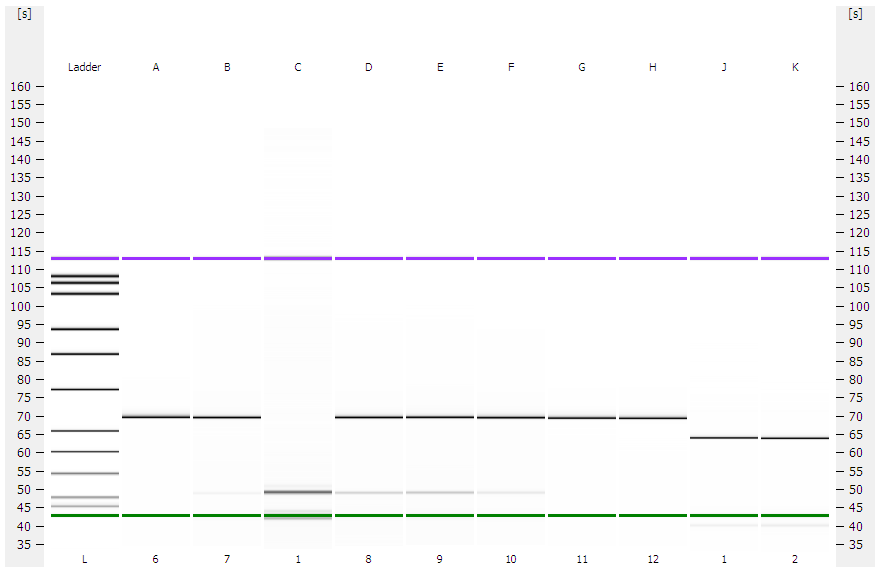

Supplement: Figure S1 — Gel Plot of H3 and H1-seasonal Spiked Samples. Displays the generated gel plot for the series of spiked samples A-K. Samples A, B, D, E, F, G and H display the 230 bp amplicon for the H3 subtype. Samples J and K display the 179 bp amplicon for the H1 seasonal subtype. Sample C is a no template control, and the 50 bp amplicon is a primer dimer associated with the H3 primers. This primer dimer is also visible in some of the lower concentration H3 samples, but it does not interfere with the sub-typing process. (TIF) [file pone.0073497.s001.tif]
