# Supplementary material for: Microdroplet Sandwich Real-Time RT-PCR for Detection of Pandemic and Seasonal Influenza Subtypes
Source: PLoS One. 2013 Sep 16;8(9):e73497. doi: 10.1371/journal.pone.0073497 (PMC3774678; doi:10.1371/journal.pone.0073497)
Supplement: File S1 — Spiked sample validation of off-tablet controls. (DOCX) [file pone.0073497.s004.docx]

# Microdroplet sandwich RT-PCR for detection of pandemic and seasonal influenza subtypes

*Stephanie L. Angione^1^, Zintis Inde^2^, Christina M. Beck^1^, Andrew W. Artenstein^3^, Steven M. Opal^4,5^, Anubhav Tripathi^1*^*

**SUPPORTING INFORMATION**

**SUPPORTING METHODS S1:**

**Spiked sample validation of off-tablet controls:** For initial validation of the assay primers, each primer set was tested individually against a set of 10 spiked influenza virion samples of unknown concentration and subtype in respiratory sample collection media, provided by Memorial Hospital of Rhode Island. The corresponding gel plot in figure S1 displays the positive results for the primer pairs, and no cross-reactivity was found between primer sets. Samples A, B, D, E, F, G and H were all validated as the H3 subtype and were demonstrated to be H1- seasonal and swine negative. Sample C was determined to be influenza negative and samples J and K were H1 seasonal positive. Again, samples J and K were found to have no reactivity with swine or H3 primer pairs. Additionally, after determining the subtype results of each of the samples, the key was provided to demonstrate the sensitivity of the H3 primer set, as samples A-H were provided as serially diluted virions. From the range of concentrations provided in the spiked samples, the results indicated that we were able to detect viral RNA as low as 10^2^ copies/mL for the H3 primer pair.
