# Supplementary material for: Microdroplet Sandwich Real-Time RT-PCR for Detection of Pandemic and Seasonal Influenza Subtypes
Source: PLoS One. 2013 Sep 16;8(9):e73497. doi: 10.1371/journal.pone.0073497 (PMC3774678; doi:10.1371/journal.pone.0073497)
Supplement: Supplementary file 3 [file pone.0073497.s002.docx]

Memorial Hospital Clincal Samples												
I.D.	PCR	Subtype	Antigen	Culture	RNA[ng/ul]		I.D.	PCR	Subtype	Antigen	Culture	RNA[ng/ul]
1	-	N/A	-	-	22.8		21	+	H3	+	+	22.2
2	-	N/A	-	-	13.2		37	+	H3	+	+	37.6
3	+	H3	-	+	24.6		38	+	H3	+	+	21.1
4	-	N/A	-	-	31.3		57	+	H3	+	+	12.3
5	-	N/A	-	-	20.3		64	+	H1p	+	+	13.6
6	-	N/A	-	-	26.2		76	+	H3	+	+	14.0
7	-	N/A	-	-	21.2		86	+	H3	+	-	22.0
8	-	N/A	-	-	21.3		87	+	H3	+	-	16.3
9	-	N/A	-	-	20.2		88	+	H3	+	-	45.5
10	-	N/A	-	-	19.5		89	+	H1p	+	+	22.5
11	-	N/A	-	-	24.6		90	+	H1p	+	-	40.6
12	-	N/A	-	-	21.0		91	+	H3	+	+	19.6
13	-	N/A	-	-	28.3		92	+	H3	+	+	21.4
14	-	N/A	-	+	18.3		93	+	H1p	+	+	21.4
15	-	N/A	-	-	17.4		94	+	H3	+	+	38.7
16	+	H3	-	+	17.8		95	+	H3	+	+	52.4
17	-	N/A	-	-	40.1		96	+	H3	+	-	47.9
18	-	N/A	-	-	33.0		97	+	H3	+	-	44.4
19	-	N/A	-	-	33.1		98	+	H1p	+	+	36.3
20	-	N/A	-	-	36.8		99	+	H3	+	+	38.8
