# Supplementary material for: Microdroplet Sandwich Real-Time RT-PCR for Detection of Pandemic and Seasonal Influenza Subtypes
Source: PLoS One. 2013 Sep 16;8(9):e73497. doi: 10.1371/journal.pone.0073497 (PMC3774678; doi:10.1371/journal.pone.0073497)
Supplement: Supplementary file 4 [file pone.0073497.s003.docx]

Sample #	Ct	Copy Number<br>log RNA copies/mL
88	23.4	6.8
89	29.2	5.1
93	31.9	4.3
95	18.3	8.3
97	19.8	7.8
